# Identifying COVID-19 cases and extracting patient reported symptoms from Reddit using natural language processing

**DOI:** 10.1038/s41598-023-39986-7

**Published:** 2023-08-22

**Authors:** Muzhe Guo, Yong Ma, Efe Eworuke, Melissa Khashei, Jaejoon Song, Yueqin Zhao, Fang Jin

**Affiliations:** 1 Department of Statistics, George Washington University, 2121 I St NW, Washington, DC 20052 USA; 2 Office of Biostatistics, Office of Translational Sciences, Center for Drug Evaluation and Research, Food and Drug Administration (FDA), 10903 New Hampshire Avenue, Silver Spring, MD 20993 USA; 3 Epidemiology and Drug Safety, IQVIA Real World Solutions, Durham, USA; 4 Division of Epidemiology II, Office of Pharmacovigilance and Epidemiology, Office of Surveillance and Epidemiology, Center for Drug Evaluation and Research, Food and Drug Administration (FDA), 10903 New Hampshire Avenue, Silver Spring, MD 20993 USA

**Keywords:** Data mining, Machine learning, Statistical methods, Public health, Applied mathematics, Computer science, Statistics, SARS-CoV-2

## Abstract

We used social media data from “covid19positive” subreddit, from 03/2020 to 03/2022 to identify COVID-19 cases and extract their reported symptoms automatically using natural language processing (NLP). We trained a Bidirectional Encoder Representations from Transformers classification model with chunking to identify COVID-19 cases; also, we developed a novel QuadArm model, which incorporates Question-answering, dual-corpus expansion, Adaptive rotation clustering, and mapping, to extract symptoms. Our classification model achieved a 91.2% accuracy for the early period (03/2020-05/2020) and was applied to the Delta (07/2021–09/2021) and Omicron (12/2021–03/2022) periods for case identification. We identified 310, 8794, and 12,094 COVID-positive authors in the three periods, respectively. The top five common symptoms extracted in the early period were coughing (57%), fever (55%), loss of sense of smell (41%), headache (40%), and sore throat (40%). During the Delta period, these symptoms remained as the top five symptoms with percent authors reporting symptoms reduced to half or fewer than the early period. During the Omicron period, loss of sense of smell was reported less while sore throat was reported more. Our study demonstrated that NLP can be used to identify COVID-19 cases accurately and extracted symptoms efficiently.

## Introduction

Early studies of COVID-19 symptoms focused on clinical symptoms derived from patients hospitalized or clinically treated^1–4^. However, the utility of these data is limited as many patients either do not seek healthcare intervention or are not treated due to the mild nature of their disease. Additionally, studies have shown that the clinical sequelae of SARS‐CoV‐2 infection are highly variable^5^, and dependent on disease severity; therefore, adequate capture of the broad spectrum of COVID-19 disease severity will be useful in understanding the natural course of the disease, from early detection through healthcare intervention and treatment. With the changing SARS‐CoV‐2 viral variants landscape, there is also a need to understand the differences in symptomatology to ensure continued surveillance and adopt appropriate treatment strategies. Social media platforms are viable sources of patient information since they are avenues for patients to freely discuss their symptoms and these data are easily accessible. Murray et al. analyzed personal narratives from COVID-19 positive Reddit users using topic modelling and sentiment analysis, providing a perspective on COVID-19 patient experiences when mental health issues co-occur^6^. Sarker et al.^7^ extracted and analyzed self-reported COVID-19 symptoms on Twitter, and Sarker et al.^8^ mined Reddit to discover long-COVID symptoms self-reported by users. Khashei et al.^9^ also manually analyzed the patient-reported COVID-19 experiences from Reddit data early in the pandemic. These studies required labor intensive manual annotations for case identification and symptom extraction and could be costly and time-consuming to implement for future COVID periods. Thus, we explored whether natural language processing (NLP) modeling could automate the manual process and facilitate systematic rapid COVID-19 case identification and symptom extraction. We aimed to identify COVID-19 positive cases from the Reddit data using an automated NLP model, followed by extracting symptoms of COVID-19 positive cases using another set of NLP models. We plan to evaluate whether self-reported symptoms change over time as the wildtype SARS‐CoV‐2 virus and early dominant variants mutated to the Delta and then the Omicron variants.

In this study, we present a novel method to identify COVID-19 positive cases and automatically extract symptoms from the Reddit community “r/covid19positive”. First, we developed a classification model using Bidirectional Encoder Representations from Transformers (BERT)^10^ to determine whether authors tested positive based on their posts and verified the model using labels generated by manual review. Then, we used the BERT^10^ and its biomedical version BioBERT^11^ question answering (multi-answer) based method to roughly extract symptoms, where non-relevant words may be captured and multiple symptoms may not be separated. To refine these symptoms, we developed a novel dual-corpus expansion method to collect keywords and related words from the named symptoms retrieved. This expansion method uses GoogleNews-vectors-negative300 as word embedding^12–14^, which is based on the semantics of words rather than word strings to automatically expand the corpora. The extracted symptoms were clustered using a newly developed semantic-based adaptive clustering method named adaptive rotation clustering (ARC), inspired by k-means clustering^15,16^. Finally, using automatic mapping function based on keywords and word frequency, we mapped the clustered symptoms to the Unified Medical Language System (UMLS)^17^ to obtain the symptom standard names. Notably, we developed this method for the automated processing of original unlabeled posts to final extracted COVID-19 symptoms according to UMLS standard names without any manual intervention.

## Data

The data for this study consisted of posts and comments from the COVID-19 forum on the freely available social media platform Reddit (www.reddit.com). We identified a user-generated topic-specific forum on the Reddit named /r/covid19positive and downloaded data by an R package called redditr which can interface with the Pushshift.io Reddit API^18^. The downloaded data contained posts and comments from three periods (March 2020 to May 2020, July 2021 to September 2021, and December 2021 to March 2022) with distinctive prevalent SARS-CoV-2 strains. Our data set contains information publicly available on Reddit and the data is generally already anonymized. Therefore, we have no identifying information for any of the authors to begin with. Additionally, to the best of our knowledge, there were no instances of authors providing Protected Health Information (PHI) that would lead to any kind of reasonable identification. We have complied with the relevant Reddit’s terms, user agreement, and conditions on data mining and user privacy.

We defined the SARS-CoV-2 early period as March 2020 to May 2020 and extracted 31,337 pieces of data (1626 posts and 29,711 comments). Each author was manually labeled as COVID-19 positive or non-positive (including COVID-19 negative, commenters only, and unconfirmed cases) based on all their posts and comments. This annotation process was performed by a single annotator, relying on explicit author reported confirmation that they received a positive COVID-19 test result, and the details are published in^9,19^. The dataset was used for two tasks: (i) Developing a classification model to identify COVID-19 positive Reddit authors. (ii) Developing a comprehensive NLP model to extract COVID-19 symptoms from positive authors' posts. We defined the Delta variant dominant period (referred to as the Delta period) from July 2021 to September 2021 during which we downloaded 119,655 pieces of data and the Omicron variant dominant period (referred to as the Omicron period) from December 2021 to March 2022 during which we downloaded 132,037 pieces of data. The Delta and Omicron variant dominant periods were not used for model development, but only for model application.

## Methods

### BERT classification model

**Figure 1:**
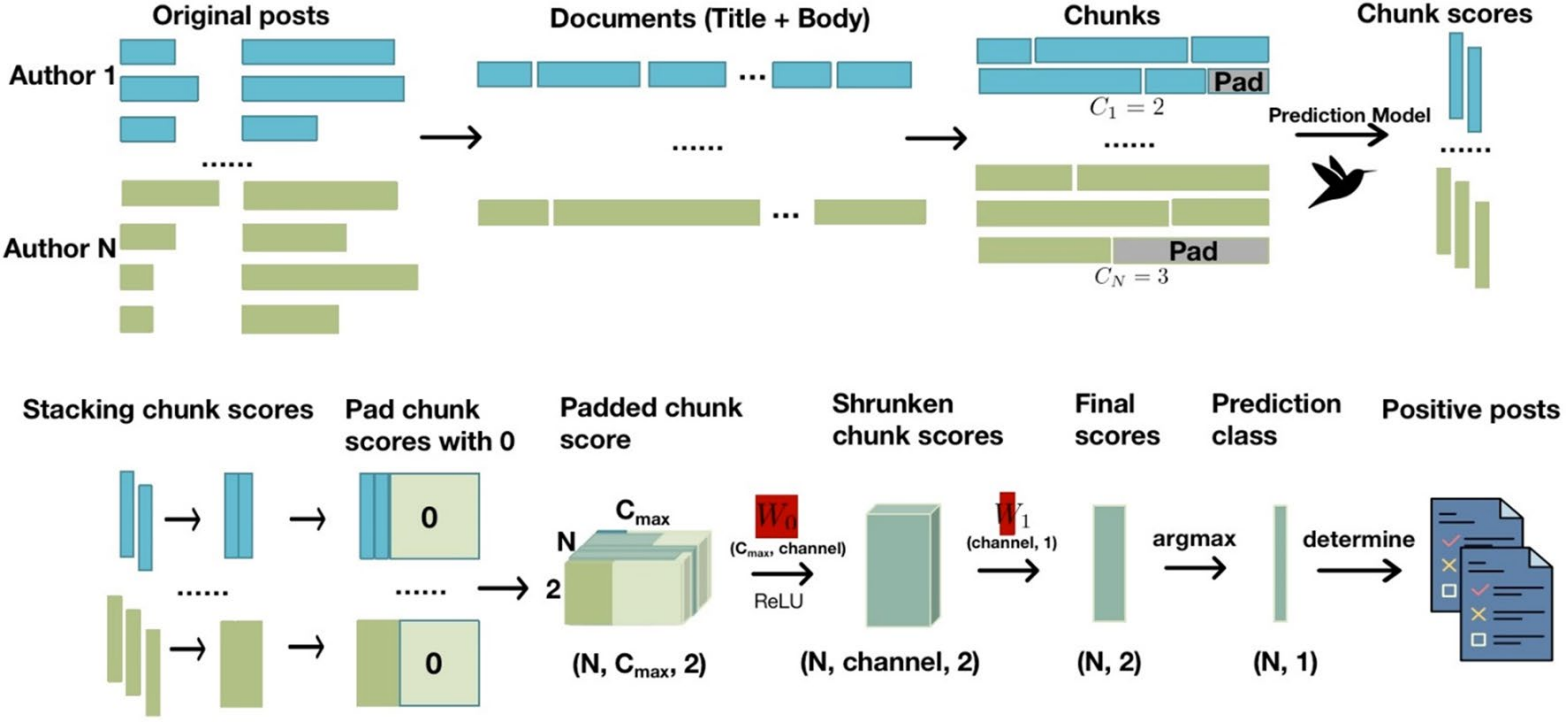
Classification model overview. The first row shows that we convert the original posts into chunks and feed them into the BERT-Large for Sequence Classification model to get chunk scores. The second row shows that the weights of chunk scores are trained with deep neural networks, and the final prediction is obtained by weighting chunk scores.

The classification model process is illustrated in Fig. 1. We manually reviewed and labeled 681 authors from the SARS-CoV-2 early period into two categories (COVID-19 Positive, COVID-19 non-positive) based on their 31,331 pieces of data with six pieces removed due to unassigned labels. We randomly divided the data into training and test sets in the ratio of 7:3. To categorize authors rather than posts/comments, for each author, we combined all the posts and comments in a stacked fashion to form an author-level “document”. We split longer documents into chunks of 512 tokens and padded shorter documents with placeholders to 512 tokens. Then, we fed chunks into the BERT-Large Sequence Classification model, resulting in two-dimensional (probability of COVID-19 Positive and COVID-19 non-positive) chunk scores. The multiple chunk scores from a single author were used to predict the author's final classification. Because the number of chunk scores varies by authors, we pre-specified a fixed number of chunk scores as 100. For authors with fewer chunk scores, we padded with chunk score 0; for authors with more chunk scores, we truncated the extra ones. A three-dimensional box of N (number of authors), ${C}_{max}$ (number of chunk scores), and 2 (probability of COVID-19 Positive and COVID-19 non-positive) was created to feed into a deep neuron network model. A weight matrix ${W}_{0}$ (${C}_{max}$, channel) was used to convert the (N, ${C}_{max}$, 2) matrix to the (N, channel, 2) matrix. A second weight matrix ${W}_{1}$ (channel, 1) was used to predict the COVID-19 Positive and COVID-19 non-positive probability. The weight matrices ${W}_{0}$ and ${W}_{1}$ were trained together and combined with a rectified linear unit (ReLU) activation function to make the final prediction. The configuration of parameters in the classification model can be found in Table 1 of the Supplementary Information File.

We illustrate the process using one author as an example. the ${i}^{th}$ author originally has c_i_ chunk scores: $${S}_{i1}= \begin{array}{cc}\left({p}_{11}^{(i)}\right.& \left.{p}_{12}^{(i)}\right)\end{array}$$, $${S}_{i2}= \begin{array}{cc}\left({p}_{21}^{(i)}\right.& \left.{p}_{22}^{(i)}\right)\end{array}$$,…, $${S}_{i{c}_{i}}= \begin{array}{cc}\left({p}_{{c}_{i}1}^{(i)}\right.& \left.{p}_{{c}_{i}2}^{(i)}\right)\end{array}$$, where ${p}_{j1}^{(i)}$ and ${p}_{j2}^{(i)}$ represent the probability that the ${j}^{th}$ chunk of the ${i}^{th}$ author belongs to class 1 (COVID-19 non-positive) and class 2 (COVID-19 positive), respectively. We padded or truncated the chunk scores to ${C}_{max}$ (100) rows to get the padded chunk score ${S}_{i}$:

1 $${S}_{i}= \left(\genfrac{}{}{0pt}{}{\begin{array}{c}{S}_{i1}\\ \vdots \\ {S}_{i{c}_{i}}\end{array}}{\begin{array}{c}0\\ \vdots \\ 0\end{array}}\right)=\left(\begin{array}{cc}\begin{array}{c}{p}_{11}^{(i)}\\ \vdots \\ {p}_{{c}_{i}1}^{(i)}\end{array}& \begin{array}{c}{p}_{12}^{(i)}\\ \vdots \\ {p}_{{c}_{i}2}^{(i)}\end{array}\\ \begin{array}{c}0\\ \vdots \\ 0\end{array}& \begin{array}{c}0\\ \vdots \\ 0\end{array}\end{array}\right)$$

Using ${W}_{0}$ and ${W}_{1}$, the final prediction class was obtained by the non-linear combination of the weighted chunk scores as following:

2 $${y}_{i}= \underset{\mathit{index }\in \{1, 2\}}{\mathrm{argmax}}{W}_{1}^{T}ReLU({W}_{0}^{T}{S}_{i})$$

Having both ${W}_{0}$ and ${W}_{1}$, the model allows different weights on the final prediction incorporating the authors’ posting order. To evaluate the accuracy of our classification method, we (1) calculated the prediction accuracy on the early-period dataset with gold labels. (2) tested the correlation between the number of positive authors we identified and the number of cases in the US reported to the CDC (https://covid.cdc.gov/covid-data-tracker/#datatracker-home).

### NLP symptom extraction model: QuadArm

**Figure 2:**
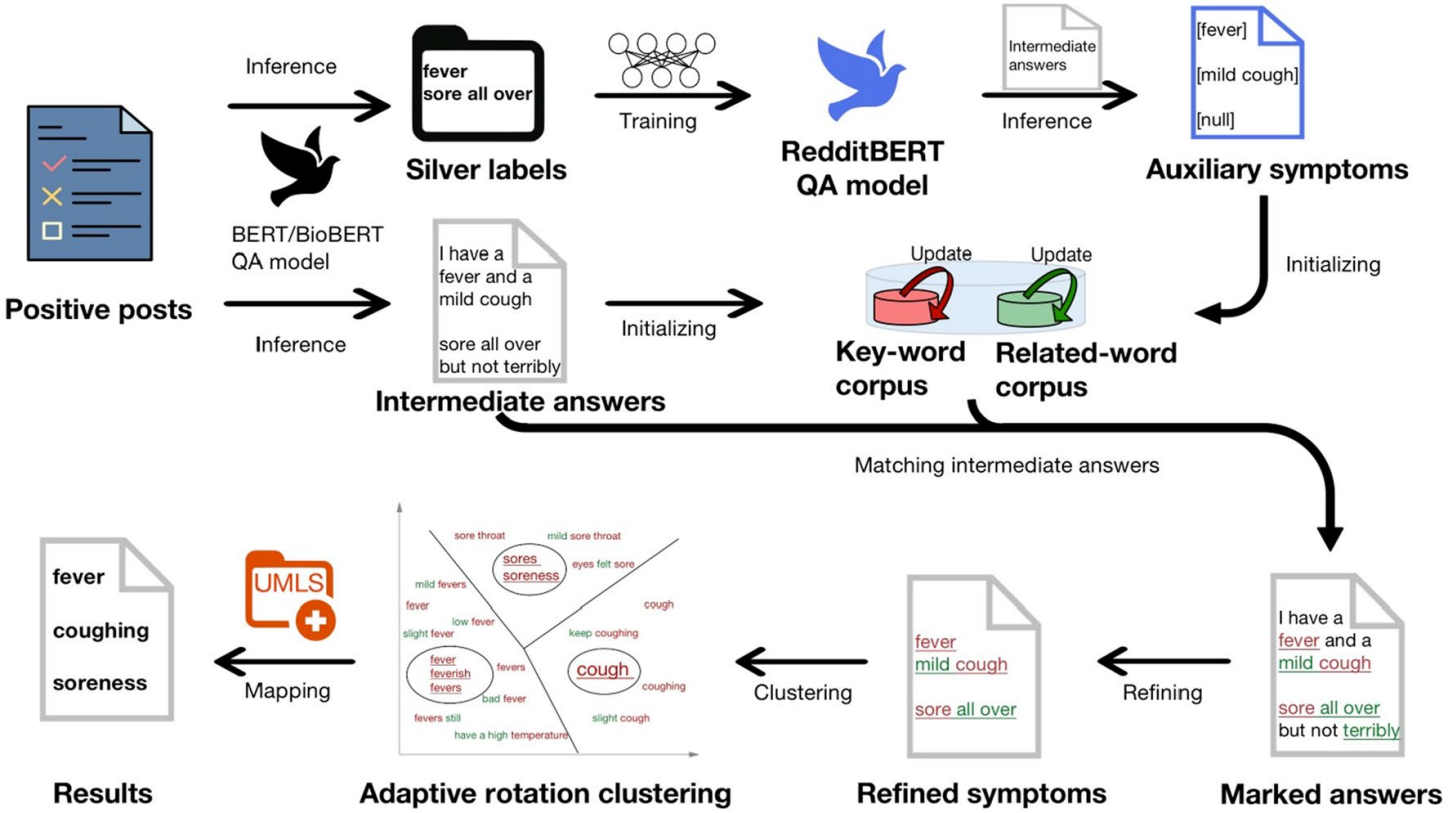
QuadArm model overview. Model inputs are positive posts and outputs are symptoms named by the UMLS. The model consists of four steps: BERT/BioBERT for question answering, dual-corpus expansion, adaptive rotation clustering, and mapping.

In the second task, we built a comprehensive NLP model to extract symptoms from the posts of COVID-19 positive authors. This model consists of four consecutive steps: (i) BERT/BioBERT for Question-Answering, (ii) dual-corpus expansion, (iii) adaptive rotation clustering (ARC), and (iv) mapping to various symptoms in the UMLS system. Figure 2 shows the overview diagram of this model. We describe the role and construction of these methods in detail.

#### BERT/BioBERT for question-answering

BERT^10^ is one of the state-of-the-art language models that can perform NLP tasks including question answering, and BioBERT^11^ is a language model developed later under the BERT framework specifically for the biomedical field. We started with the BERT and later added the BioBERT. Since there was no gold standard label for COVID-19 symptoms, we extracted symptoms without pre-labeling the posts. Considering the diversity and colloquiality of the symptom expressions in the Reddit data, we found a way to automatically recognize which words in a post indicate “symptoms” using the BERT/BioBERT for Question Answering (QA) model. Note that we could not simply apply the QA model directly to the Reddit posts to get the final symptoms for the following reasons: first, each post did not always contain only one symptom but may contain multiple symptoms or no symptoms. Second, if an author reported several symptoms in a sentence, we needed to extract these distinct symptoms rather than several symptoms in one description (e.g., “fever” and “cough”, rather than “I had a fever with a mild cough”). Third, BERT/BioBERT for general QA tasks is not fine-tuned by social media data, resulting in some linguistic features of Reddit difficult to be identified accurately. Thus, the answers expected from the QA model are not exact, but rough candidate answers which can be refined later. The complete configuration of parameters utilized for the QA step in the QuadArm model can be referenced in Table 2 of the Supplementary Information File. However, it is worth noting that appropriate adjustments of parameters are also permissible.

*QA for getting Intermediate Answers:* To ensure the direct collection of symptoms from the authors themselves, beyond those merely mentioned in the post, we formulated the question of the BERT/BioBERT QA models as “What are my uncomfortable symptoms?” and treated each post as a passage. The maximum length of each question-passage pair was set to 300 tokens. For passages longer than this threshold we separated them into smaller passages and allowed 128 tokens to overlap between two adjacent smaller passages. We then applied fine-tuned BERT/BioBERT models on the positive posts to obtain the Intermediate Answers. The details of obtaining Intermediate Answers are as follows: Firstly, we obtained multiple rough answers for each post by setting the following thresholds:

3 $$\left\{\begin{array}{c}{Score}_{start}>0\\ {Score}_{end}>0\\ {Score}_{start}+ {Score}_{end}>2\end{array}\right.$$

where the Start Scores Score_start_ and End Scores Score_end_ can be found in BERT^10^ for details. Note that the higher the thresholds, the more formal the answers obtained, and the fewer the number. Thus, we used relatively low thresholds to get more candidate Intermediate Answers. Suppose we currently get an answer set $\mathscr{A}$ with $m$ answers for one post:

4 $$\mathscr{A}=\left\{{\alpha }_{1},{\alpha }_{2},\dots ,{\alpha }_{m}\right\}.$$

To prevent excessive overlap between multiple answers, we use the Levenshtein distance (edit distance)^20,21^ to calculate distances between answers. The Levenshtein distance between the answer ${\alpha }_{i}$ with length ${|\alpha }_{i}|$ and the answer ${\alpha }_{j}$ with length ${|\alpha }_{j}|$ is given by:

5 $$Lev\left({\alpha }_{i},{\alpha }_{j}\right)=\left\{\begin{array}{c} \begin{array}{cc}{|\alpha }_{i}|& if {|\alpha }_{j}|=0\\ {|\alpha }_{j}|& if {|\alpha }_{i}|=0\end{array}\\ Lev\left(t\left({\alpha }_{i}\right),t\left({\alpha }_{j}\right)\right) if {\alpha }_{i}^{1}={\alpha }_{j}^{1} \\ 1+min\left\{Lev\left(t\left({\alpha }_{i}\right),{\alpha }_{j}\right),Lev\left({\alpha }_{i},t({\alpha }_{j})\right) , \left.Lev\left(t\left({\alpha }_{i}\right),t({\alpha }_{j})\right)\right\} \right., otherwise\end{array}\right.$$

where $t(x)$ is the string of all but the first character of $x$, and ${x}^{1}$ is the first character of the string $x$. Then, the Levenshtein distance ratio between ${\alpha }_{i}$ and ${\alpha }_{j}$ is defined as:

6 $${r}_{Lev}\left({\alpha }_{i},{\alpha }_{j}\right)= \frac{Lev\left({\alpha }_{i},{\alpha }_{j}\right)}{Alignment\, length\, of \left({\alpha }_{i},{\alpha }_{j}\right) }$$

If ${r}_{Lev}\left({\alpha }_{i},{\alpha }_{j}\right)>0.8$, we discard the shorter answer of ${\alpha }_{i}$ and ${\alpha }_{j}$ to avoid losing the extracted information. The current answers we got are called the Intermediate Answers which needs further refinement. The lower the Levenstein ratio threshold, the more answers with duplicate text will be removed. We used 0.8 to retain more Intermediate Answers going into next steps. Mathematically, the intermediate answer set $\Omega$ for one post satisfies:

7 $$\left\{\begin{array}{c}\Omega \subseteq \mathscr{A}\\ {r}_{Lev}\left({\omega }_{i},{\omega }_{j}\right)\le 0.8, \forall {\omega }_{i},{\omega }_{j}\in \Omega \\ {|\omega }_{i}|>{|\alpha }_{j}|, \forall {\omega }_{i}\in \Omega , {\alpha }_{j}\in \mathscr{A}, \text{and } {r}_{Lev}\left({\omega }_{i},{\alpha }_{j}\right)>0.8\end{array}\right.$$

We further identified the symptoms embedded in the Intermediate Answers through the dual-corpus expansion method.

*QA for getting Auxiliary Symptoms:* To run the dual-corpus expansion model, we needed Auxiliary Symptoms to initialize the word corpus. Ancillary Symptoms are the accurate symptoms capturing the linguistic characteristics of Reddit and may not include all symptoms reported. To extract Auxiliary Symptoms, we trained a RedditBERT QA model to adapt the model to the language features of Reddit using silver labels. The silver labels were obtained using the same approach as obtaining Intermediate Answers, but with more stringent thresholds: replacing 2 with 6 in Eq. (3); replacing 0.8 with 0.6 in Eq. (7). Next, we treated the Intermediate Answers as short passages and implement the RedditBERT QA model with the question “What are my uncomfortable symptoms?”. The strictest thresholds were set in Eq. (3) by setting the sum of ${\mathrm{Score}}_{\mathrm{start}}$ and ${\mathrm{Score}}_{\mathrm{end}}$ greater than 10. We did not use Levenshtein distance ratio to remove similar answers, but for each intermediate answer we kept the top three scoring answers (Empty answers may be used if the number of valid answers was < 3) which became the Auxiliary Symptoms. These Auxiliary Symptoms, along with the Intermediate Answers, can help initialize the word corpus in the dual-corpus expansion method in the subsequent steps.

#### Dual-corpus expansion method

The dual-corpus expansion method was designed to obtain two COVID-19 symptom corpora, namely COVID-19 key-word corpus (abbrev. ${\mathrm{Corpus }}_{k}$) and COVID-19 related-word corpus (abbrev. ${\mathrm{Corpus }}_{r}$). The corpora will mark the words in the Intermediate Answers as keywords, related words or null, and allow them to be refined later. This method initialized corpora using some trustworthy words and then checked every word in the Intermediate Answers to iteratively update the corpora until their sizes no longer changed. The initialized words of the corpora come from two sources: (1) If an Auxiliary Symptom containing less than three words matches the corresponding Intermediate Answer, then the words in this Auxiliary Symptom will be used. (2) The words in eleven COVID-19 symptoms given by CDC (https://www.cdc.gov/coronavirus/2019-ncov/symptoms-testing/symptoms.html) will be directly used. Note that the Stopwords^22^ providing no useful symptom information will be excluded from the corpora. Whether a word will initialize ${\mathrm{Corpus }}_{k}$ or ${\mathrm{Corpus }}_{r}$ is determined manually: the words representing symptoms themselves, such as "fever" and "cough" will initialize ${\mathrm{Corpus }}_{k}$. and the words only having modifying or associative roles with symptoms, such as "mild" and "loss" will initiate ${\mathrm{Corpus }}_{r}$. The initialized words in our model are shown in Table 1.

**Table 1:** Initial COVID-19 key-word corpus and initial COVID-19 related-word corpus.

Initialized keywords (72 words)	Initialized related words (33 words)
ache, aches, achy, allergies, belching, bloating, blood, body, brain, breath, breathe, breathing, bronchitis, burping, canker, chest, chills, confusion, congestion, cough, coughing, cramps, dehydrated, dehydration, depressing, diarrhea, dizziness, dizzy, exhaustion, fatigue, fatigued, fever, fevers, gi, hangover, headache, headaches, hyperacidity, inflammation, insomnia, muscle, nausea, nauseous, nose, pain, pains, pneumonia, pressure, reflux, runny, sinus, sleeping, smell, sneezing, sore, sores, spleen, stomach, supraventricular, sweats, swelling, tachycardia, taste, throat, tightness, tiredness, tonsillitis, ulcers, voice, vomiting, vomiting, weakness	anxiety, bad, difficulty, dry, feeling, fingertips, fog, getting, grade, helplessness, issues, light, losing, loss, low, medium, mild, of, overworking, really, red, rough, scared, seasonal, severe, shortness, small, sob, spells, stinks, streaks, symptoms, weird

We update the two corpora based on the semantic relevance of the words. So, we first applied a word embedding called Google-News-vectors-negative300^12–14^ to convert words into numerical vectors, then used dual-corpus expansion (see Algorithm 1 in the Supplementary Information File) to update ${\mathrm{Corpus }}_{k}$ and ${\mathrm{Corpus }}_{r}$. The final key-word corpus consists of 397 words the final related-word corpus consists of 1933 words.

#### Adaptive rotation clustering method

After getting the two corpora, we labeled the words in the Intermediate Answers as keywords (red) and related words (green) as illustrated in Fig. 2. Subsequently, we applied the following criteria to refine the marked answers and derive the term referred to as the “refined symptom”: Each refined symptom should (1) be composed entirely of words from the two corpora, (2) contain at least one word from the key-word corpus, (3) be the longest consecutive phrase formed by the words in the two corpora in the single Intermediate Answer. Next, due to the diversity of expression of refined symptoms, we developed a novel clustering method ARC (see Algorithm 2 in the Supplementary Information File) to semantically cluster refined symptoms. The ARC is an iterative process where we cluster the keywords and refine symptoms rotationally until the clusters converge. In the end, each cluster of the ARC output will have a “key” (i.e., keyword(s) from the key-word corpus) and a “value” (i.e., a group of refined symptoms). The final number of clusters does not need to be specified in advance because it is determined adaptively by the iterative algorithm.

#### Mapping method

After ARC, we had clusters of symptoms consisting of synonyms and imprecise descriptions. To standardize and professionalize symptom names, we mapped the clusters to the UMLS, a repository of biomedical vocabularies developed by the US National Library of Medicine. This will also help to estimate the symptom frequencies. In UMLS, there is a glossary called Concept Unique Identifier (CUI), which assigns a unique identifier to each concept that belongs to a particular terminology, making strings with the same meaning linked to it. The mapping method was implemented by matching the frequent words, keywords, and symptoms in refined symptoms clusters sequentially with the unique identifier in CUI dictionary until a match was found. Clusters with no match (rarely found) were deleted.

### Symptom analysis

We conducted statistical tests to compare the symptoms extracted using our NLP models to the previously reported symptoms in literature and symptoms in different COVID-19 variant periods. We used the Chi-square test of independence^23^ to compare our extracted symptoms with those extracted by Sarker et al.^7^ in the early period. We conducted the Pearson correlation test^24^ to quantify the synchrony between the daily number of official COVID-19 cases from CDC and the daily number of cases we extracted. To exclude the influence of the day (for example, fewer cases were usually reported on weekends), seven-day average moving window was implemented on all the series. We also performed two-sample Kolmogorov–Smirnov test^25^ to examine whether the distribution of number of symptoms we extracted differ during the Delta and the Omicron periods. Finally, we used a Z-test to compare the differences in proportions of co-appearing symptoms between the two variant periods.

### Ethical approval

The research activities in the manuscript are determined NOT to be human subjects research. This is consistent with the FDA’s standard operating Procedure on “Determining Whether an Activity is Human Subjects Research” by the Office of the Commissioner, published on February 19, 2019, as well as the guidance in the following link. https://www.hhs.gov/ohrp/regulations-and-policy/guidance/research-involving-coded-private-information/index.html. During our work, there is no interaction with the individuals posted on Reddit and all data were completely de-identified.

## Results

In this section, we present the main results of the classification model and the symptom extraction QuadArm model. The COVID-19 case identification results are shown in Section “COVID-19 cases identification”, followed by the statistics on symptoms in Section “COVID-19 symptoms extraction”. Through visualization, the number of symptoms and the statistical comparison of the symptoms in the three periods are presented in Section “Symptom diversity and trends”. Then, the similarities and differences of symptoms in the two variant periods and the co-appearance of symptoms are discussed in Section “Comparison of symptoms and co-appearance of symptoms in the two later variant periods”. In Section “COVID-19 symptom corpus system”, we display the COVID-19 symptom corpus system obtained by our framework through a visual presentation.

### COVID-19 cases identification

For the SARS-CoV-2 early period, there were 681 authors who posted and 310 were labelled as positive and the rest were labeled as non-positive through a manual post-by-post review. The BERT classification model was trained and achieved good performance metrics on the test dataset, including an accuracy of 91.2%, precision of 91.0%, recall of 91.5%, and an f1-score of 91.2%. Using this model, we identified 8794 and 12,094 positive authors for the Delta and Omicron periods, respectively. The symptom extraction was later implemented in the posts of positive authors.

**Figure 3:**
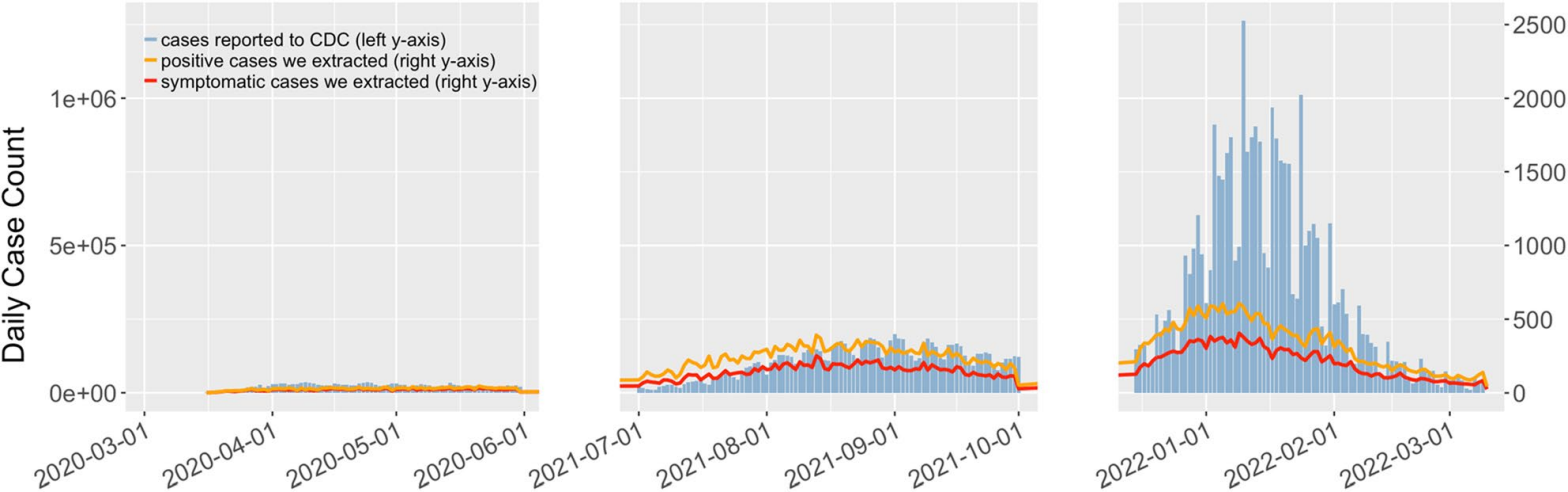
Daily trends in number of COVID-19 cases reported to the CDC and we extracted, for the corresponding three periods.

Figure 3 shows the trends in number of COVID-19 cases in the US reported to CDC as well as number of COVID-19 cases we extracted in the three periods. In addition to the positive cases, we also reported symptomatic cases, defined as positive cases with at least one symptom extracted by our QuadArm model, further discussed in Sections “COVID-19 symptoms extraction” through “COVID-19 symptom corpus system”. To provide a clear contrast to the substantial number of officially reported cases, we used an alternative y-coordinate (right y-axis) with a smaller scale for our classified positive cases and symptomatic cases. The trend of positive and symptomatic cases we identified showed high correlation with the officially reported cases (Pearson’s r = 0.560 and 0.600 for the Early period, Pearson’s r = 0.809 and 0.834 for the Delta period, and Pearson’s r = 0.867 and 0.895 for the Omicron period). This finding supports the soundness of our approach to identifying cases. Although the Pearson correlation for Early period is relatively weak compared to the Delta and Omicron periods, this phenomenon may be due to inadequate reporting of both early cases to the CDC and posts on social media. Details of the synchrony test results can be found in Test 1 in the Supplementary Information File.

### COVID-19 symptoms extraction

For each COVID-19 variant period, we developed two symptom extraction models (QuadArms), using BERT and BioBERT respectively for the QA step and obtained two sets of results presented in Table 2. Results from Sarker et al. in the early COVID-19 period are also listed in Table 2 for direct comparison. Number of authors with symptoms, symptoms, and symptom frequencies are summarized by BERT/BioBERT model and COVID-19 variant periods. For the SARS-CoV-2 early period, QuadArm with BERT extracted symptoms from 288 authors and QuadArm with BioBERT extracted symptoms from 294 authors. Overall, the frequencies of symptoms from the two models were very similar except for inflammation (2.1%, 20.7%). The top five most common symptoms were coughing (53.5%, 56.5%), fever (52.8%, 54.8%), loss of sense of smell (39.9%, 40.5%), headache (38.9%, 40.1%), and sore throat symptom (37.5%, 39.8%). For the Delta period, 4589 and 5110 out of 8794 positive authors reported symptoms based on our two models. Compared to SARS-CoV-2 early period, all symptoms were reported notably less. The proportion of authors with loss of sense of smell (21.7%, 21.3%) was almost equal to that with coughing (23.8%, 24.0%) and fever (21.3%, 23.2%). For Omicron period, 7080 and 7573 out of 12,094 positive authors reported symptoms. Compared to Delta period, sore throat (30.6%, 29.8%) was reported more frequently and loss of sense of smell (12.1%, 11.6%) was reported less frequently.

**Table 2:** Comparison of COVID-19 common symptoms reported on Reddit positive authors at different time periods.

	Our study (BERT) Early period	Our study (BioBERT) Early period	Sarker et al Study on Twitter^7^	Our study (BERT) Delta period	Our study (BioBERT) Delta period	Our study (BERT) Omicron period	Our study (BioBERT) Omicron period
Date from to	03.14.2020 05.31.2020	03.14.2020 05.31.2020	02.01.2020 04.14.2020	07.01.2021 10.01.2021	07.01.2021 10.01.2021	12.15.2021 03.10.2022	12.15.2021 03.10.2022
Number of authors classified as positive	310	310	–	8794	8794	12,094	12,094
Number of authors with symptom extracted	288	294	169	4589	5110	7080	7573
Symptom	n (n/288)	n (n/294)	n (n/169)	n (n/4589)	n (n/5110)	n (n/7080)	n (n/7573)
Coughing	154 (53.5%)	166 (56.5%)	99 (58.6%)	1092 (23.8%)	1225 (24.0%)	1886 (26.6%)	2110 (27.9%)
Fever	152 (52.8%)	161 (54.8%)	113 (66.9%)	979 (21.3%)	1183 (23.2%)	1379 (19.5%)	1630 (21.5%)
Loss of sense of smell	115 (39.9%)	119 (40.5%)	49 (29.0%)^#^	994 (21.7%)	1086 (21.3%)	856 (12.1%)	875 (11.6%)
Headache	112 (38.9%)	118 (40.1%)	64 (37.9%)	863 (18.8%)	927 (18.1%)	1406 (19.9%)	1491 (19.7%)
Sore throat symptom	108 (37.5%)	117 (39.8%)	41 (24.3%)^◊^	827 (18.0%)	868 (17.0%)	2164 (30.6%)	2258 (29.8%)
Dyspnea	98 (34.0%)	106 (36.1%)	62 (36.7%)	209 (4.6%)	230 (4.5%)	271 (3.8%)	292 (3.9%)
Pain	100 (34.7%)	105 (35.7%)	73 (43.2%)^⊕^	502 (10.9%)	526 (10.3%)	1044 (14.7%)	1085 (14.3%)
Lack of energy	92 (31.9%)	104 (35.4%)	72 (42.6%)^Ψ^	633 (13.8%)	703 (13.8%)	991 (14.0%)	1069 (14.1%)
Chest discomfort	89 (30.9%)	103 (35.0%)	–	279 (6.1%)	312 (6.1%)	464 (6.6%)	488 (6.4%)
Stops breathing	43 (14.9%)	101 (34.4%)	–	139 (3.0%)	394 (7.7%)	138 (1.9%)	504 (6.7%)
Aches	85 (29.5%)	91 (31.0%)	73 (43.2%)^⊕^	520 (11.3%)	571 (11.2%)	968 (13.7%)	1064 (14.0%)
Tired	56 (19.4%)	61 (20.7%)	–	325 (7.1%)	419 (8.2%)	410 (5.8%)	530 (7.0%)
Inflammation	6 (2.1%)	61 (20.7%)	–	21 (0.5%)	374 (7.3%)	34 (0.5%)	434 (5.7%)
Chill	57 (19.8%)	61 (20.7%)	43 (25.4%)	319 (7.0%)	371 (7.3%)	575 (8.1%)	646 (8.5%)
Chest pain	50 (17.4%)	50 (17.0%)	39 (23.1%)	137 (3.0%)	132 (2.6%)	206 (2.9%)	206 (2.7%)
Common cold	40 (13.9%)	49 (16.7%)	–	367 (8.0%)	556 (10.9%)	599 (8.5%)	840 (11.1%)
Diarrhea symptom	53 (18.4%)	49 (16.7%)	15 (8.9%)	177 (3.9%)	200 (3.9%)	225 (3.2%)	241 (3.2%)
Nausea	46 (16.0%)	44 (15.0%)	19 (11.2%)	280 (6.1%)	277 (5.4%)	463 (6.5%)	451 (6.0%)
Dizziness	47 (16.3%)	43 (14.6%)	15 (8.9%)^Δ^	221 (4.8%)	213 (4.2%)	369 (5.2%)	363 (4.8%)

#Reported as Anosmia in this study.

◊Reported as Oropharengeal pain (sore throat) in this study.

⊕Pain and Aches are combined together and reported as Body ache & general pain in this study.

ΨReported as Fatigue in this study.

∆Reported as Dizziness/disorientation/confusion in this study.

Because Sarker et al. and we both extracted the COVID-19 symptoms from social media data (Twitter for Sarker et al. Reddit for us) in a similar early period (February 01, 2020 to April 14, 2020 for Sarker et al. March 14, 2020 to May 31, 2020 for us), we compared whether the extracted symptom frequencies were consistent. We performed Chi-square test of independence for the 14 common symptoms in Table 2. We found that there is no statistically significant difference (*p* > 0.05) for the following 9 symptoms: coughing (56.5% vs 58.6%), headache (40.1% vs 37.9%), dyspnea (36.1% vs 36.7%), pain (35.7% vs 43.2%), lack of energy (35.4% vs 42.6%), chill (20.7% vs 25.4%) chest pain (17.0% vs 23.1%), nausea (15.0% vs 11.2%), and dizziness (14.6% vs 8.9%). However, five symptoms showed statistically significant differences (*p* ≤ 0.05): fever (54.8% vs 66.9%), loss of sense of smell (40.5% vs 29.0%), sore throat (39.8% vs 24.3%), aches (31.0% vs 43.2%), and diarrhea (16.7% vs 8.9%). Differences in these five symptoms most likely were attributed to different rules of extraction methods, different social media platforms, or slight differences in the time periods. When Bonferroni's correction is applied to correct for multiple comparisons, only sore throat shows significant differences (*p* = 0.001). Details of the comparisons can be found in Test 2 in the Supplementary Information File.

### Symptom diversity and trends

In this section, we show the extracted symptom clusters and the trend of symptoms over time for the SARS-CoV-2 early period, Delta period, and Omicron period.

**Figure 4:**
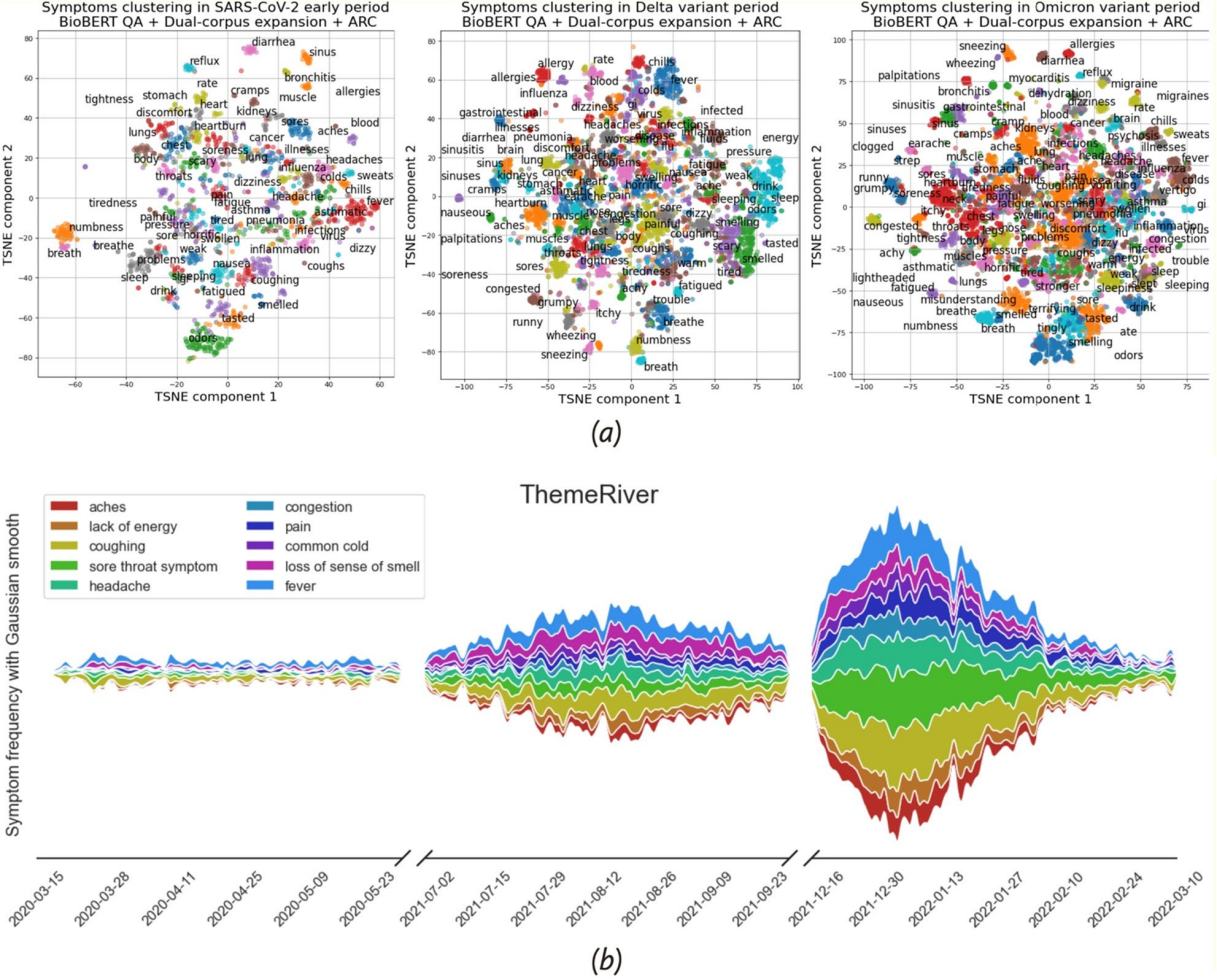
Symptom clustering and trending based on our model QuadArm with BioBERT. Panel (**a**) shows the clustering results of COVID-19 symptoms through t-SNE visualization for the SARS-CoV-2 early period, Delta period, and Omicron period, respectively. Panel (**b**) is a ThemeRiver plot showing the change of ten common symptom frequencies over time in the three COVID-19 periods.

In Fig. 4, panel (a) are clustering results of the three periods from our ARC approach using t-Distributed Stochastic Neighbor Embedding (t-SNE)^26^, which is a non-linear unsupervised technique primarily used in high-dimensional data visualization. The total number and the diversity of symptoms increase from the SARS-CoV-2 early period to the Delta period and then to the Omicron period. Panel (b) is the ThemeRiver plot showing the changes in raw counts of symptoms over time. The width of the ribbon reflects the number of authors reporting the corresponding symptom. The ribbons were smoothed with Gaussian smoothing along the date to show the clearer trends. As shown, while we observed steady raw counts in the SARS-CoV-2 early period, there were large reports of symptoms counts later which appeared to coincide with the increasing number of cases in the US.

### Comparison of symptoms and co-appearance of symptoms in the two later variant periods

In this section, we provide a visual comparison of the proportion of authors reporting the top 15 symptoms during the Delta and Omicron periods, with a statistical test for difference in the distribution of symptoms between the two periods. We also discuss the co-occurrence of symptoms for each period, as well as a statistical test for difference in co-occurrence between the two periods.

**Figure 5:**
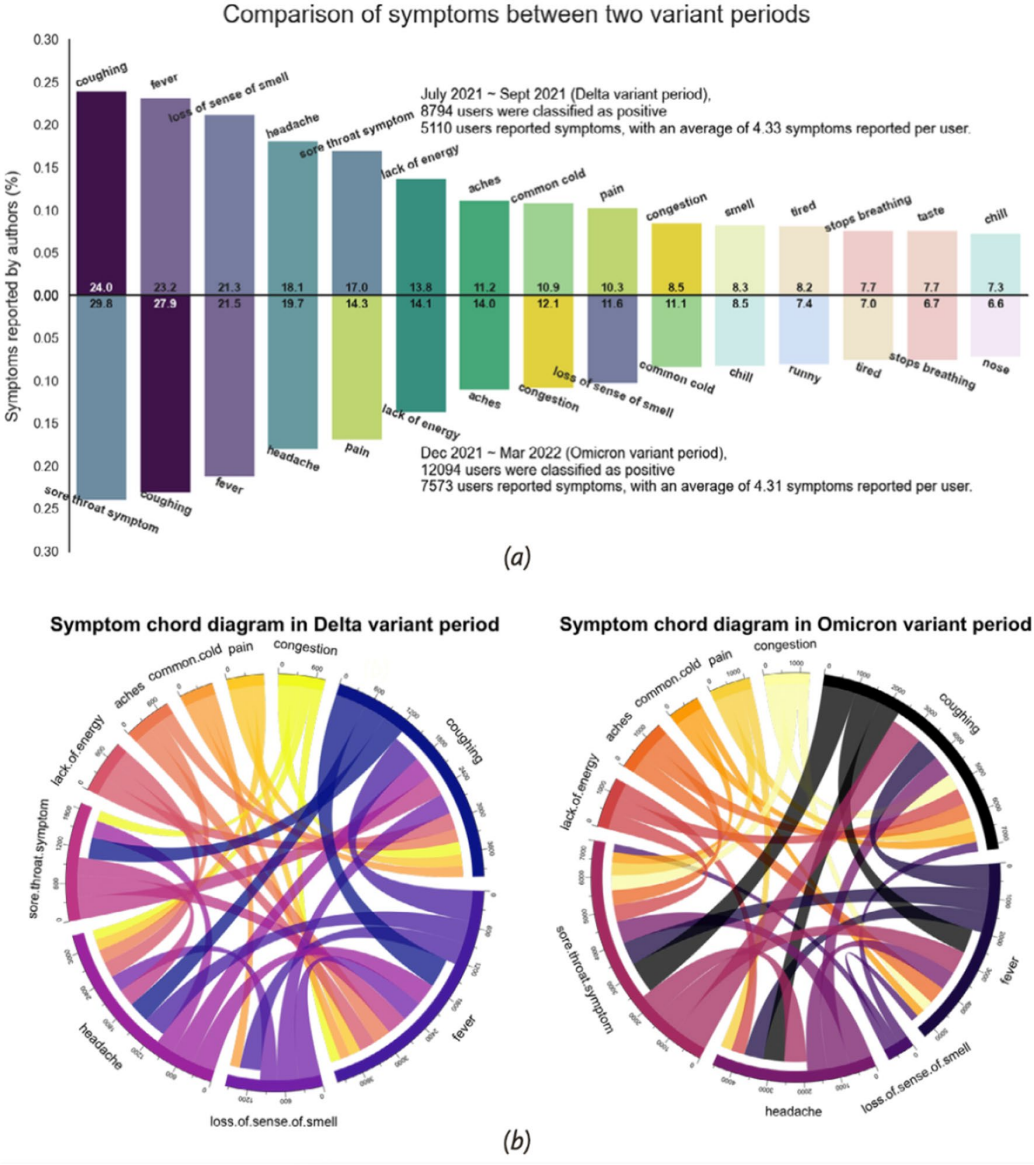
Comparison of symptoms extracted by our model (QuadArm with BioBERT) in the two virus variation periods. Panel (**a**) shows the top 15 commonly reported symptoms for each period. Panel (**b**) includes two Chord diagrams showing the co-appearance relationship between symptoms for each period. The width of the connection between two symptoms represents the number of authors with both symptoms.

Panel (a) of Fig. 5 shows that throughout the two COVID-19 variant periods, authors commonly reported fever, cough, and headache more frequently than other symptoms. There are notable changes in symptoms between the two variant periods. During the Delta period, 17.0% of authors reported sore throat symptoms, compared with 29.8% in Omicron period. This moved the sore throat from being the fifth most common symptom during the Delta period to the most common symptom during the Omicron period. Meanwhile, a significant decrease in authors with loss of sense of smell was observed (21.3% before, 11.6% after).

In the Delta period, authors reported an average of 4.33 (± standard deviation 4.51) different symptoms; in the Omicron period authors reported 4.31 (± standard deviation 4.44) symptoms. The two distributions were not statistically significantly different (two-sample Kolmogorov–Smirnov test p value = 0.817). See Test 3 in Supplementary Information File for more details.

Panel (b) of Fig. 5 includes two Chord Diagrams representing co-appearance of the top ten symptoms. The width of the ribbon between two symptoms represents the number of authors who reported both symptoms and therefore the “correlation” between the two symptoms. For simplicity, we only show three other symptoms that co-appear with each symptom. For example, for both Delta and Omicron periods, most authors who reported coughing also reported fever, headache, or sore throat. During the Delta period, generally, the top three co-appearance symptoms were coughing, fever, and headache, but during the Omicron period, sore throat replaced headache.

We further tested the difference in proportions of co-appearance symptoms of each symptom between the two variant periods. The two-proportion Z-test was performed for each pair of the ten common symptoms shown in Panel (b) of Fig. 5. The probability of any two symptoms appearing simultaneously was not significantly different between the two periods. Although sore throat replaced headache in the top three co-appearance symptoms, this difference was not statistically different. The complete test results can be found in Test 4 in the Supplementary Information File.

### COVID-19 symptom corpus system

**Figure 6:**
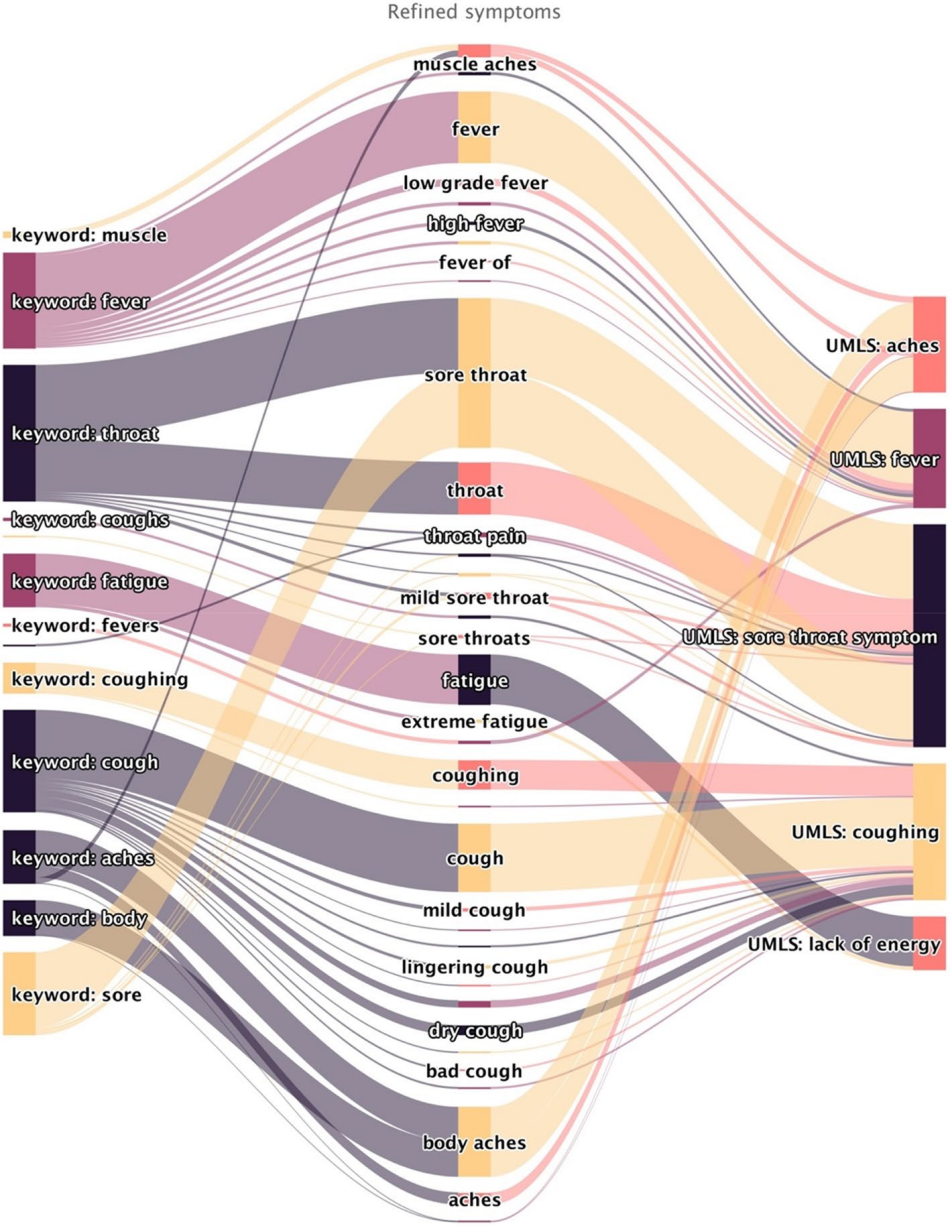
A closer look of the COVID-19 symptom corpus system (Omicron variant period). The left column is the COVID-19 key-word corpus obtained from our dual-corpus expansion method. The middle column is refined symptoms after applying the key-words corpus on the marked answers. The right column is the final standardize medical symptom names obtained by mapping to UMLS. The width of the connection line represents the number of corresponding authors.

Figure 6 provides a closer look at our COVID-19 symptom corpus system. The thickness of the lines represents the frequency of the refined symptoms reported. The figure also demonstrates the mapping from the key-word corpus to the final UMLS. For example, the refined symptom “muscle aches” includes two keywords “muscle” and ‘aches’ in the key-word corpus, and it is mapped to the ‘ache’ symptom in UMLS along with other refined symptoms such as ‘body aches’, ‘minor body aches’ and ‘aches’.

## Discussion

In this study, we examined three COVID-19 variant dominant time periods spanning a total of nine months and collected 22,569 posts and 260,460 comments from 41,494 authors. We developed a BERT classification model to identify the COVID-19 positive authors. We also developed a QuadArm NLP framework to extract symptoms reported by the COVID-19 positive authors. Our main findings and contributions are: (1) Our classification model demonstrates good performance in identifying COVID-19 positive authors. (2) Our QuadArm NLP framework can directly extract accurate symptoms from posts without manual annotation. Our dual-corpus expansion and ARC methods are novel and successfully strengthened the model performance. (3) Our mapping from the refined symptoms to UMLS is autonomous without any manual intervention. (4) We compared symptoms reported during the SARS-CoV-2 early, Delta and Omicron periods and analyzed the pattern of symptom reporting over time. (5). We compared and tested our results against the results of published studies, as well as the results coming from official CDC reports, indirectly demonstrating the applicability of our approach.

In Table 2, the SARS-CoV-2 early period results are compared with Sarker et al.^7^ study on Twitter over a similar period. The top five most common symptoms extracted by Sarker et al. on Twitter are fever (66.9%), coughing (58.6%), body ache & general pain (43.2%), fatigue (42.6%) and headache (37.9%). In our result, pain (34.7%, 35.7%) and aches (29.5%, 31.0%) are two separate symptoms but combined by Sarker et al. Fatigue is a symptom reported by Sarker et al. mostly likely captured by lack of energy instead in our models. Our study revealed that, in the SARS-CoV-2 early period, symptoms reported by Reddit authors were similar to those reported on Twitter. For the top five most frequently reported symptoms, both social media platforms reported coughing, fever, and headache. In addition, Reddit authors reported loss of sense of smell and sore throat and Twitter authors reported body ache & general pain and fatigue. Part of the reason of the difference may be that in our model, ache and pain are considered two separate symptoms.

We found that although the three COVID-19 variant dominant periods were each about three month long, the authors, posts, and comments increased remarkably in the latter two periods, possibly because more people were infected during these two periods. For the three periods, coughing, fever, headache, and sore throat were in the top five most frequently reported symptoms. Loss of sense of smell was in the top five in the first two periods but was replaced by pain in the Omicron period. During the Omicron period, loss of sense of smell became less common, and sore throat and pain were more common, consistent with the community study published in the Lancet^27^.

We also found a decrease in the percentage of authors reporting symptoms in the later variant periods compared to the early period, a phenomenon could be explained by two factors: (1) The effect of COVID-19 vaccination (the shorter presentation of symptoms) as more people were vaccinated in the latter two periods. (2) A surge of users on the “r/covid19positive” subgroup, many posting infrequently and reporting only a small number of symptoms.

There are four notable differences between symptoms found in social media and in the clinical setting: (1) social media is flooded with irrelevant information. (2) The descriptions of symptoms can be more complex and varied compared to clinical descriptions from initiation to resolution. (3) Symptoms reported on social media are often less severe than clinical symptoms because patients who go to the hospital may have more severe symptoms. (4) The age distribution of patients is different. According to Reddit Usage Statistics (https://thrivemyway.com/reddit-statistics/), people between the age 18 and 29 years were Reddit's largest user base (64%) and only 7% of Reddit users are over 50 years. Clinical studies usually include older patients. A CDC study of COVID-19 symptom profiles^28^ included 52% of persons aged $\ge$ 65 years. In the WHO report^29^, when COVID-19 disease was first characterized, most patients (77.8%) were aged 30 to 69 years. Therefore, symptom analysis on social media may make up for the lack of younger patients and mild to moderate symptoms in clinical studies.

There are inherent limitations to symptom extraction and analysis on social media. (1) The extracted symptoms, including symptom names and the reported time of appearance, are unverifiable since they were self-reported. This inherent reliance on self-reporting may introduces the potential for bias in the subsequent analysis. (2) Some categories of reported symptoms overlapped (e.g., chest pain/difficulty breathing or pain/aches). Symptoms may appear over or under-reported if not captured in anticipated categories. (3) Misspellings, grammatical errors, and even crude abbreviations of words or phrases in the post can make the model unable or incorrectly extract symptoms. (4) A lack of standardization—this is an issue both in capturing patient demographic information and being forced to rely on spontaneous communication of symptoms as opposed to a survey that has targeted questions. In addition, because our model was trained using the data of SARS-CoV-2 early period and derived symptoms in the key-word corpus were fixed, our model may not recognize new symptoms that first occurred during the latter two variant periods. Still, our key-word corpus contains almost 400 key words, corresponding to over 300 symptoms, thus the inability to capture symptoms would only have occurred if a new, unexpected symptom occurred. In the future, we plan to train our QuadArm model with more data covering longer COVID-19 prevalence periods to have a more exhaustive symptom list.

Furthermore, there exists potential for advancing and broadening the scope of our approach through the integration of complementary methods and techniques. For instance, accounting for the diverse communication preferences among different subreddit groups^30^ or incorporating research on emotion-related content within the Reddit platform^31^ could help mitigate biases in symptom extraction. Additionally, enhancing the model through the utilization of multi-question QA techniques^32^ holds promise for achieving higher precision in symptom identification. In cases where adequate human resources and annotated training data are available, alternative approaches such as employing named entity recognition (NER) methods^33^ to replace the QA step or implementing pre-screening to target the extraction scope^34^ can be explored.

## Conclusion

In this study, we demonstrated that NLP models can be used to identify COVID-19 positive patients and extract COVID-19 symptoms without any subsequent manual annotation. The model accuracies were further validated by their high synchrony with the number of cases and symptoms reported by the CDC and other research. Our COVID-19 symptom corpus system not only provides a final list of symptoms (e.g., fever) but also a distribution of variations of symptoms (e.g., fever, high fever, low grade fever). These autonomous models can be easily applied to later time periods in the future without manual annotations. Although this approach was created using the social media data, the methodology can be easily adapted to larger, more systematic, and credible data sources such as electronic health records (EHR). As feature generation from unstructured data is a key step in utilizing EHR, we believe our work in this area bring us one step closer to fully utilizing the fast-accumulating health data timely and accurately.

### Supplementary Information

Supplementary Information.

## Data Availability

The data that support the findings of this study are available from Melissa Khashei on reasonable request. The code that supports the findings of this study is available from Muzhe Guo on reasonable request.
