# Supplementary material for: Identifying COVID-19 cases and extracting patient reported symptoms from Reddit using natural language processing
Source: Sci Rep. 2023 Aug 22;13:13721. doi: 10.1038/s41598-023-39986-7 (PMC10444846; doi:10.1038/s41598-023-39986-7)
Supplement: Supplementary file 1 — Supplementary Information. [file 41598_2023_39986_MOESM1_ESM.pdf]

Supplementary Information File

Identifying COVID-19 cases and extracting patient reported symptoms from Reddit using natural language processing

Muzhe Guo[1], Yong Ma[2], Efe Eworuke[3†], Melissa Khashei[4], Jaejoon Song[2], Yueqin Zhao[2], and Fang Jin[1*]

[1]Department of Statistics, George Washington University, 2121 I St NW, Washington, DC, 20052 USA
[2]Office of Biostatistics, Office of Translational Sciences, Center for Drug Evaluation and Research, Food and Drug Administration (FDA), 10903 New Hampshire Avenue, Silver Spring, MD, 20993, USA
[3]Epidemiology and Drug Safety, IQVIA Real World Solutions
[4]Division of Epidemiology II, Office of Pharmacovigilance and Epidemiology, Office of Surveillance and Epidemiology, Center for Drug Evaluation and Research, Food and Drug Administration (FDA), 10903 New Hampshire Avenue, Silver Spring, MD, 20993, USA

[†]Efe Eworuke was an employee of the Food and Drug Administration during the time of study conduct. She is now employed by IQVIA.
[*]Corresponding author: Fang Jin

Supplementary table 1: Configuration of parameters in classification model

**Sequence classification training**	
pretrained model	BERT large
maximum sequence length	512
learning rate	2e-5
adam epsilon	1e-8
batch size	32
number of epochs	2
**Weight matrix training**	
model	Deep Neuron Network
maximum chunk number (C_{max})	100
learning rate	3e-3
batch size	544
number of epochs	200
channel dim	64
output dim	2

Supplementary table 2: Configuration of parameters of QA step in QuadArm model

**Obtaining Intermediate Answers (IA) / Silver Labels (SL)**	
finetuned model	BioBERT/BERT large
maximum input length	300
maximum overlap length	128
$Score_{start}$ threshold	0
$Score_{end}$ threshold	0
$Score_{start}$+$Score_{end}$ threshold	2 (IA) / 6 (SL)
Levenshtein ratio	0.8 (IA) / 0.6 (SL)
top n answer	N/A
**RedditBERT QA model training**	
pretrained model	BioBERT/BERT large
learning rate	3e-5
batch size	16
number of epochs	2
weight decay	0.01
**Obtaining Auxiliary Symptoms**	
finetuned model	RedditBERT QA model
maximum input length	50
maximum overlap length	25
$Score_{start}$ threshold	0
$Score_{end}$ threshold	0
$Score_{start}$+$Score_{end}$ threshold	10
Levenshtein ratio	N/A
top n answer	3

Note: As QA score thresholds increase and the Levenshtein ratio threshold decreases, the number of retained answers will decrease, but the formality will increase. The above thresholds can be adjusted based on the noise level of the text and the specific requirements of the task. For example, in cases where the text is clean or the answers are intended for accurate labeling purposes, higher QA score thresholds and a lower Levenshtein ratio threshold are recommended.

Supplementary algorithm 1: dual-corpus expansion

Algorithm 1 Dual-corpus Expansion

Input: Initialized $\mathbf{Corpus}_{k0} = \{\boldsymbol{w_1}, \boldsymbol{w_2}, \dots, \boldsymbol{w_{n_0}}\}$, Initialized $\mathbf{Corpus}_{r0} = \{\boldsymbol{w'_1}, \boldsymbol{w'_2}, \dots, \boldsymbol{w'_{n'_0}}\}$, Stopword set $\boldsymbol{S}$, and confidence level factor $\boldsymbol{\alpha}$.

Output: $\mathbf{Corpus}_k$ and $\mathbf{Corpus}_r$.

Algorithm:

1. Calculate pairwise cosine similarities in $\mathbf{Corpus}_{k0}$ and $\mathbf{Corpus}_{r0}$, get $\mathbf{X}_0 = \{\boldsymbol{x_1}, \boldsymbol{x_2}, \dots, \boldsymbol{x_{n_0(n_0-1)/2}}\}$, and $\mathbf{Y_0} = \{\boldsymbol{y_1}, \boldsymbol{y_2}, \dots, \boldsymbol{y_{n'_0(n'_0-1)/2}}\}$, respectively.
2. Assume both $\mathbf{X}_0$ and $\mathbf{Y_0}$ are i.i.d. sample sets and identify their probability density functions (pdf): $\boldsymbol{x_1}, \boldsymbol{x_2}, \dots, \boldsymbol{x_{n_0(n_0-1)/2}} \sim \boldsymbol{f_k(x)}$ and $\boldsymbol{y_1}, \boldsymbol{y_2}, \dots, \boldsymbol{y_{n'_0(n'_0-1)/2}} \sim \boldsymbol{f_r(y)}$.
3. Denote quantile threshold function as $\boldsymbol{q^*(n)} = \left(\frac{\boldsymbol{1-\alpha}}{\boldsymbol{n-1}}\right)^{\boldsymbol{1/n}}$.
4. Initialize $\boldsymbol{i = 0}$. Repeat:
   - Obtain a new word $\boldsymbol{w^i}$, $\boldsymbol{w^i \notin S}$.
   - Calculate the cosine similarity of $\boldsymbol{w^i}$ to each word in $\mathbf{Corpus}_{ki}$ and $\mathbf{Corpus}_{\mathbf{ri}}$ and get cosine similarity samples: $\boldsymbol{X_i^w} = \{\boldsymbol{x_1^w}, \boldsymbol{x_2^w}, \dots, \boldsymbol{x_{n_i}^w}\}$ and $\boldsymbol{Y_i^w} = \{\boldsymbol{y_1^w}, \boldsymbol{y_2^w}, \dots, \boldsymbol{y_{n'_i}^w}\}$.
   - Find the largest order statistic in $\boldsymbol{X_i^w}$ and $\boldsymbol{Y_i^w}$, denoted by $\boldsymbol{x_{(n_i)}^w}$ and $\boldsymbol{y_{(n'_i)}^w}$.
   - if $\boldsymbol{x_{(n_i)}^w} > \boldsymbol{q^*(n_i)}$ quantile of $\boldsymbol{f_k(x)}$:

     $\mathbf{Corpus}_{ki} \leftarrow \mathbf{Corpus}_{ki} \cup \{\boldsymbol{w^i}\}$.

     else if $\boldsymbol{y_{(n'_i)}^w} > \boldsymbol{q^*(n'_i)}$ quantile of $\boldsymbol{f_r(y)}$:

     $\mathbf{Corpus}_{ri} \leftarrow \mathbf{Corpus}_{ri} \cup \{\boldsymbol{w^i}\}$.

     else:

     Continue.
   - $\boldsymbol{i} = \mathbf{i + 1}$.

   Until both $\mathbf{Corpus}_{ki}$ and $\mathbf{Corpus}_{ri}$ have stable sizes.
5. $\mathbf{Corpus}_k \leftarrow \mathbf{Corpus}_{ki}$; $\mathbf{Corpus}_r \leftarrow \mathbf{Corpus}_{ri}$.

In Algorithm 1, we set the confidence level factor $\alpha = \mathbf{0.05}$. Using a variant of the SINDy algorithm,[1] step 2 detected that $\boldsymbol{f_k}(x)$ follows a Beta distribution (To make $\mathbf{Corpus}_k$ more tightly gathered, we only used upper half samples of $\mathbf{X}_0$) and $f_r(x)$ follows a Burr distribution.

Supplementary algorithm 2: adaptive rotation clustering (ARC)

Mathematically, suppose there are n keywords in the key-word corpus denoted by $w_1, w_2, \dots, w_n$, and we have m refined symptoms denoted by $s_1, s_2, \dots, s_m$. All the keywords and the refined symptoms have been transformed into numerical vectors using the average of the hidden layers of the BERT-Base.[2] After implementing the ARC algorithm that uses cosine similarity as semantic distance, we obtained G clusters of refined symptoms with size $m_1, m_2, \dots, m_G$, respectively. Finally, we removed symptom clusters of size one, because upon examination we found that such clusters did not contain symptoms but strange words.

Algorithm 2 Adaptive Rotation Clustering (ARC)

Input: Key-word corpus: $\{\boldsymbol{w_1}, \boldsymbol{w_2}, \dots, \boldsymbol{w_n}\}$, Refined symptoms: $\{\boldsymbol{s_1}, \boldsymbol{s_2}, \dots, \boldsymbol{s_m}\}$

Output: $\boldsymbol{G}$ refined symptom clusters: $\left\{\left(\left\{\boldsymbol{w_j}: \boldsymbol{j} \in \boldsymbol{J_k}\right\}: \{\boldsymbol{s_i}: \boldsymbol{i} \in \boldsymbol{I_k}\}\right): \boldsymbol{k} = \mathbf{1}, \mathbf{2}, \dots, \boldsymbol{G}\right\}$, where $\boldsymbol{I_1}, \boldsymbol{I_2}, \dots, \boldsymbol{I_G}$ are $\boldsymbol{G}$ disjoint sets of symptom index with size $\boldsymbol{m_1}, \boldsymbol{m_2}, \dots, \boldsymbol{m_G}$ such that $\cup_{\boldsymbol{i}=\mathbf{1}}^{\boldsymbol{G}} \boldsymbol{I_i} = \{\mathbf{1}, \mathbf{2}, \dots, \boldsymbol{m}\}$. $\boldsymbol{J_1}, \boldsymbol{J_2}, \dots, \boldsymbol{J_G}$ are $\boldsymbol{G}$ disjoint sets of keyword index with size $\boldsymbol{n_1}, \boldsymbol{n_2}, \dots, \boldsymbol{n_G}$ without constraints on their union.

Algorithm:

1. Use $\left\{\boldsymbol{w_j}: \boldsymbol{j} = \mathbf{1}\right\}, \left\{\boldsymbol{w_j}: \boldsymbol{j} = \mathbf{2}\right\}, \dots, \left\{\boldsymbol{w_j}: \boldsymbol{j} = \boldsymbol{n}\right\}$ as the initialized keyword groups, where each group contains only one word. And the initialized Key Centroid (denoted by $\boldsymbol{C_k}$) of the $\boldsymbol{k}$-th keyword group is $\boldsymbol{w_k}$ itself.

   Use $\{\boldsymbol{s_i}: \boldsymbol{i} = \mathbf{1}\}, \{\boldsymbol{s_i}: \boldsymbol{i} = \mathbf{2}\}, \dots, \{\boldsymbol{s_i}: \boldsymbol{i} = \boldsymbol{m}\}$ as the initialized refined symptom clusters, where each cluster contains only one symptom. And the initialized Value Centroid (denoted by $\boldsymbol{C_v}$) of the $\boldsymbol{k}$-th refined symptom cluster is $\boldsymbol{s_k}$ itself. So, there are $\boldsymbol{G} = \boldsymbol{m}$ refined symptom clusters for the initialization.

2. Initialize $\mathbf{t} = \mathbf{0}$. Repeat:
   - Update the symptom clusters by assigning each symptom cluster to the closest Key Centroid in $\boldsymbol{C_k}$. Remove the keyword groups and Key Centroids with no symptoms.
   - Recompute the centroid of each symptom cluster to update Value Centroid set $\boldsymbol{C_v}$.
   - Update the keyword groups by assigning each keyword group to the closest Value Centroid in $\boldsymbol{C_v}$.
   - Recompute the centroid of each keyword group to update Key Centroid set $\boldsymbol{C_k}$.
   - Merge symptom clusters as well as the corresponding keyword groups if the cosine similarity of their Value Centroid > $\mathbf{max}\,\{\mathbf{100}\% - \boldsymbol{t} \times \mathbf{0.5}\%,\ \mathbf{95}\%\}$.
   - $\mathbf{t} = \mathbf{t} + \mathbf{1}$

   Until the symptom clusters do not change.

Test 1: Pearson's correlation coefficient between the number of COVID-19 cases we extracted and the number of COVID-19 cases reported to CDC

Pearson's correlation coefficient with the number of COVID-19 cases reported to CDC

	Early period		Delta period		Omicron period	
**Symptom series**	Pearson's r	P value	Pearson's r	P value	Pearson's r	P value
**Positive cases we extracted**	0.560	<0.001 (***)	0.809	<0.001 (***)	0.867	<0.001 (***)
**Symptomatic cases we extracted**	0.600	<0.001 (***)	0.834	<0.001 (***)	0.895	<0.001 (***)

Note: * indicates $p < 0.05$; ** indicates $p < 0.01$; and *** indicates $p < 0.001$

Test 2: Chi-square independent test

Results of Sarker et al. vs Our result (based on QuadArm with BioBERT model)

	**Symptom**		**Symptom**	
	Coughing	No - coughing	Fever	No - fever
**Models**				
Ours	166	128	161	133
Sarker et al.	99	70	113	56
**P - value**	0.658		0.011 (*)	
	Loss of sense of smell	No - loss of sense of smell	Headache	No - headache
**Models**				
Ours	119	175	118	176
Sarker et al.	49	120	64	105
**P - value**	0.013 (*)		0.631	
	Sore throat	No - sore throat	Dyspnea	No - dyspnea
**Models**				
Ours	117	177	106	188
Sarker et al.	41	128	62	107
**P - value**	0.001 (**)		0.892	
	Pain	No - pain	Lack of energy	No - lack of energy
**Models**				
Ours	105	189	104	190
Sarker et al	73	96	72	97
**p-value**	0.111		0.123	
	Aches	No - aches	Chill	No - chill
**Models**				
Ours	91	203	61	233
Sarker et al	73	96	43	126
**p-value**	0.008 (**)		0.244	
	Chest pain	No - chest pain	Diarrhea	No - diarrhea
**Models**				
Ours	50	244	49	245
Sarker et al	39	130	15	154
**p-value**	0.111		0.019 (*)	
	Nausea	No - nausea	Dizziness	No - dizziness
**Models**				
Ours	44	250	43	251
Sarker et al	19	150	15	154
**p-value**	0.261		0.072	

Note: * indicates $p < 0.05$; ** indicates $p < 0.01$; and *** indicates $p < 0.001$

Test 3: Two-sample Kolmogorov-Smirnov test

The Two-sample Kolmogorov–Smirnov test is used to test whether two underlying probability distributions differ. The statistic is:

$$D_{n,m} = Sup_x |F_{1,n}(x) - F_{2,m}(x)|$$

where $F_{1,n}(x)$ and $F_{2,m}(x)$ are the empirical distribution functions of the first and the second sample respectively, and Sup is the supremum function. For large samples, the null hypothesis is rejected at level α if $D_{n,m} > c(\alpha)\sqrt{\frac{m+n}{mn}}$, where n and m are the sizes of first and second sample respectively. The value of $c(\alpha)$ is given in the table below for the most common levels of α:

α	**0.1**	**0.05**	**0.01**	**0.005**	**0.001**
$c(\alpha)$	1.224	1.358	1.628	1.731	1.949

In our test, the null hypothesis is that the two distributions of number of symptoms are identical. The statistic $D_{n,m}$ = 0.0135, p-value = 0.6268.

Test 4: Comparing two proportions

Here we take the proportion of patients with cough who has fever co-appeared as an example to illustrate this two-proportion Z-test: for each of the two periods, we firstly counted the total number of authors showing cough, denoted by n_1 and n_2, respectively. We also calculated that a percentage of p_1 of these n_1 authors showed fever, while this percentage was p_2 in the n_2 authors. Then, the Z-test statistic $(p_1 - p_2)/\sqrt{(n_1p_1(1-p_1) + n_2p_2(1-p_2))/(n_1+n_2)}$ follows a normal distribution under the null hypothesis that the two percentages are the same.

Given symptom / Co-appearance symptom	aches	common cold	congestion	coughing	fever	headache	lack of energy	loss of sense of smell	pain	sore throat symptom
aches	nan	0.905	0.937	0.929	0.888	0.903	0.942	0.980	0.995	0.991
common cold	0.984	nan	0.925	0.995	0.997	0.980	0.986	0.964	0.981	0.889
congestion	0.893	0.940	nan	0.895	0.885	0.935	0.855	0.937	0.965	0.971
coughing	0.934	0.929	0.953	nan	0.982	0.953	0.932	0.986	0.974	0.995
fever	0.930	0.956	0.933	0.877	nan	0.959	0.943	0.866	0.854	0.826
headache	0.965	0.998	0.931	0.999	0.948	nan	0.974	0.955	0.912	0.910
lack of energy	0.951	0.986	0.988	0.988	0.996	0.951	nan	0.938	0.906	0.963
loss of sense of smell	0.643	0.686	0.609	0.695	0.670	0.670	0.650	nan	0.622	0.644
pain	0.950	0.911	0.971	0.948	0.945	0.960	0.966	0.993	nan	0.977
sore throat symptom	0.789	0.870	0.902	0.753	0.797	0.800	0.726	0.882	0.844	nan

References

[1] Kaheman, K., Brunton, S. L. & Kutz, J. N. Automatic differentiation to simultaneously identify nonlinear dynamics and extract noise probability distributions from data. Machine Learning: Science and Technology 3, 015031 (2022).

[2] Devlin, J., Chang, M.-W., Lee, K. & Toutanova, K. Bert: Pre-training of deep bidirectional transformers for language understanding. arXiv preprint arXiv:1810.04805 (2018).
